# Religious Concerns About COVID-19 Vaccines: From Abortion to Religious Freedom

**DOI:** 10.1007/s10943-022-01557-x

**Published:** 2022-04-11

**Authors:** D. Gareth Jones

**Affiliations:** Department of Anatomy, University of Otago, PO Box 913, Dunedin, 9054 New Zealand

**Keywords:** COVID-19, Religion, Abortion, Religious freedom, Vaccines, Vaccine hesitancy

## Abstract

In the midst of the debate about vaccines against COVID-19 and vaccine mandates, there are a surprisingly large number of concerns coming from some churches centring on the dependence of some of the vaccines on historic abortions and limitations of freedom of choice. Although the ethical significance of separation between historic abortions and the vaccines has been widely accepted by most religious authorities, the pandemic has led to renewed concern by some. The emergence of vaccine mandates, with their potential negative impact on church attendance, has led some to oppose anything that would limit freedom of choice. Within this opposition is a medley of other messages, such as lack of trust in experts and human rights violations. Some regard vaccine passports as a form of ‘medical apartheid’ or ‘therapeutic totalitarianism’, coercing people’s conscience. A countervailing perspective is provided by most church organizations that take a far more communitarian view based upon Jesus’ teachings, identification with the poor and marginalized, and public health considerations. These Christians place far greater store on science as a gift from God, medical science as a means of transforming societies for good, and the potential of vaccines to control a rampant pandemic. Flexibility in imposing vaccine mandates is essential with onus placed on protecting the vulnerable, the community, and directed by the biblical precept of love for one’s neighbour.

## Introduction

General concerns with COVID vaccines and vaccine mandates have covered diverse territory and have elicited considerable debate on a range of topics. Of these, churches’ concerns have fallen into two dominant categories. There is the well-known and well researched topic of their origin in, or association with, abortion many years ago, to a far more general concern based on restrictions to free movement within society, stemming from objections to the introduction of vaccine mandates and certificates. For some, the latter centres on potential restrictions on unvaccinated people being able to attend church services. Of these two objections, the first has been rigorously debated, especially within Roman Catholic circles. The second is more difficult to define or come to grips with, since it is mainly driven by objections to restrictions on the freedom to hold church services. Unfortunately, many aspects of these debates have spilled over into other areas that have added to the confusion by questioning scientific data and the interpretation of the available data. This includes the safety and efficacy of the available vaccines.

## Safety and Efficacy of the COVID Vaccines

The safety and efficacy of the vaccines have been rigorously assessed, having been tested in animals, and then through the three phases of clinical trials in humans. During the human clinical trials, each vaccine was tested for safety and efficacy on tens of thousands of volunteers. Clinical trials of COVID-19 vaccines were undertaken in different countries where there were high infection rates, enabling the rapid assessment of their efficacy. In the first clinical trial, the level of antibodies in the bloodstream was measured, with laboratory tests to check on how well these neutralized the COVID-19 virus. In phases two and three, the efficacy of the vaccine was tested in thousands of participants, some of whom received the vaccine whereas others received a saline placebo. The participants were closely monitored for at least two months after the second dose. The phenomenal speed of approval was made possible by governments and research funding agencies committing enormous sums of money and people to the enterprise and with considerable global sharing of data.

The general consensus among the health community is that all the vaccines in widespread use are extremely safe. Nevertheless, the reality is that no vaccine is 100% risk-free, in the way that no medical procedure is 100% risk-free. In practice, the very small element of risk has to be weighed against the very much greater risk of being infected with COVID-19. As part of the approval process of any vaccine, there has to be evidence that adverse reactions are rare and are strongly outweighed by their effective protection from disease. To date (mid-March 2022) of the order of 11 billion doses of vaccine have been given. Safety continues to be monitored through post-marketing surveillance and safety monitoring systems in numerous countries. Regular safety updates are provided to monitoring agencies in different countries and regions, through COVID data trackers. The major vaccines in use will continue to undergo the most intensive safety monitoring in history.

All possible side effects are reported and assessed. They are generally minor, with common side effects including swelling, redness and pain at the injection site, fever, headache, tiredness, muscle pain, chills and nausea (CDC, 2021). Serious safety problems are rare and depending upon the vaccine used, include anaphylaxis, blood clots and myocarditis and pericarditis. The incidence of these is very low. For example, the incidence of myocarditis following one dose of a mRNA COVID-19 vaccine is just over 2 cases per 100,000 people; most of these have been of mild or moderate severity. It is the very rare case of vaccine-induced death that makes the headlines, but tragic as this always is it has to be seen within the context of a devastating pandemic that has to date been responsible for over 6 million deaths worldwide.

The Pfizer vaccine is at least 90% effective at preventing symptomatic infection for up to 6 months after injection following the second dose. However, as evidenced by the emergence of new mutations of the virus, the scene is constantly changing, with consequences for the continuing efficacy of vaccines and the number of doses needing to be given to individuals to bolster protection.

### Christian Supporters from Within Science

Francis Collins, who until the latter part of 2021 was Director of the National Institutes of Health (NIH) in the United States, stands out as a leading scientist and staunch supporter of the Christian faith. In earlier years, he was involved in discovering the genetic basis of cystic fibrosis and then headed the international collaboration that first mapped the entire human genome. Throughout the pandemic, he has overseen the American scientific response and in particular in directing the development of a coronavirus vaccine in record time. For him, as a man of faith, what has been supreme is the importance of evidence and as he would put it a commitment to truth. As he surveyed the 700,000 white flags on Washington’s National Mall in mid-2021, signifying the death toll from the pandemic in the United States at that time, he recognized that this failure is the result of deviation from the commitment to truth on both vaccines and masks (Gershon, 2021).

It is not surprising that Collins has found it extremely disturbing to see that evangelical Christians are among America’s strongest bastions of vaccine resistance. He is repeatedly found stating that Christians are to respect scientific knowledge. In his view, the “relationship between order and beauty” raises legitimate theological questions. For Collins, the church ought to be a light set on a hill, an entity that believes in truth. He is reported to have commented: “This is a great moment for the church to say, no matter how well intentioned someone’s opinions may be, if they’re not based upon the facts, the church should not endorse them.” (Barlow, 2021).

### Vaccine resistance by conservative Christians

The resistance of some conservative Christians prompts the query of why this is the case. A detailed answer lies beyond the scope of this article, although it appears to stem from two predominant sources: distrust of science and scientists, and the emphasis on divine health and miraculous healing among Pentecostal and charismatic Christians (Fitzgerald, 2017). Although found most strongly in the United States, elements have been transplanted to many other countries. The distrust of science and scientists is seen predominantly in the biological and biomedical sciences, rather than in the physical sciences, engineering or IT domains. It is likely that this distrust emanates from equating biological science with evolutionary theory, and since the latter has to be rejected in order to maintain trust in the biblical witness, the views of biological and biomedical scientists are met with extreme caution (Alexander, 2008). While this is far removed from debates over vaccines and vaccine mandates, the misgivings about biological science in general flow over into debates in which science appears to have made a major contribution to improving human welfare. It is seen, therefore, as a science-faith dispute, in which God’s domain is being threatened by the inroads of secular science.

Reference to Francis Collins is sufficient to illustrate that there is no hegemonic religious position on acceptance of COVID vaccination and that the frequently encountered opposition is but one perspective and a far from dominant one at that. This is because it stands in contrast to prevailing accents in the church. Historically, these have included caring for the sick, following the manner in which Christ cared for the sick. There is nothing unusual in expecting the church to play its part in combatting a widespread and contagious virus, since this was very much the case with epidemics such as smallpox and the plague (McNutt, 2021). On these occasions, efforts were made by leading theologians and church leaders to respond as best they could with the knowledge and tools at their disposal, even though in some instances it was at the cost of their own safety.

Martin Luther in 1527 when confronted by a plague, stands out for his fortitude, commitment to his flock, and sheer common sense. He recognized the importance of caring for one’s neighbour, for the community, and taking the necessary steps to protect others. The important thing was to serve one’s community. He was convinced that we are obliged to assist and help others as we ourselves would like to be helped. In very practical terms, Luther urged people to take medicine, to disinfect their homes, and, if at all possible, to avoid people and places in an effort not to spread the disease (Luther, 1999).

## Objections based on historic abortions

The origin of many vaccines in cell lines stemming from abortions performed in the 1970s and 1980s is well known and has led to considerable ethical turbulence in religious circles and in some countries also in political circles (Wadman, 2020a). The debate has also led to a distinction between ‘ethical’ vaccines (where no fetal material has been used) and ‘ethically compromised’ ones (Wadman, 2020b). Detailed charts have been produced to explain how the various COVID vaccines have been developed and whether fetal cell lines have featured at some step during development (Prentice & Sander, 2021). While this issue will not be relitigated here, it features to greater or lesser degrees in many religiously affiliated discussions. While these discussions may be regarded as of interest only to those within a religious community, this is not the case, as illustrated by Giubilini and co-workers (2021) who argue that religious objections to vaccine research are unethical, irresponsible and irrational. From their standpoint, any such objections will cause harm to other people both inside and outside religious communities.

As the pandemic has persisted throughout the past 2 years, aspects of the ethical debate have become more clearly delineated, and while the abortion issue remains fraught in some quarters, its prominence has diminished in others. This is due to a more precisely defined understanding of the nature of the link between those original abortions and the necessity of having effective vaccines to quell an otherwise overwhelming public health emergency.

The crux of the issue is two-fold; whether the abortions were undertaken in order to produce the vaccines, and whether there is a temporal relationship between the two. In both instances, the answer is a categorical ‘no’. This is crucial for those who regard any abortion as an evil act. But even if the latter is granted, it becomes important to distinguish between ‘immediate and mediate’ and ‘proximate and remote’ cooperation with the immoral act. Wrapped up in these distinctions is the degree of separation between the two events, and therefore, whether those involved in vaccine production are or are not morally complicit in benefitting from the original abortion (Rudd, 2003).

### Moral Complicity

The notion of moral complicity has played a large part in ethical reasoning starting with decisions about whether one is morally complicit in using Nazi-derived material or data in contemporary research and clinical settings. Moral complicity contends that those who use material or data derived unethically in the past are themselves implicated in the original unethical practices (Jones & Whitaker, 2009). Only two responses are possible in response to this dilemma. If the moral complicity principle is accepted no use can be made of the material or data, but if it is rejected the material can be used in an ethically acceptable manner as long as certain stipulations are met. Although contentious issues remain, societies have found a way forward by ensuring that physical and conceptual separations are erected between the original ‘evil’ act and the subsequent ‘beneficial’ act in contemporary practice. Ongoing concerns revolve around whether use of tissue or data legitimizes the original abuse and thereby rewards that abuse (Max, 1989).

In the context of vaccinations, does continuing use of abortion-derived fetal cell lines bestow upon the abortions a moral legitimacy that many religious people deplore (Maher, 2002)? If it is thought that they do, the only ethical resolution is to reject any use of the cell lines already in existence and seek alternative ways of manufacturing highly effective vaccines (Pruss, 2006). This presumes that there are morally unproblematic vaccines and/or that it would be feasible to manufacture them, not just for COVID- 19 but for all the other diseases (measles, mumps, smallpox) for which vaccines have proved life-saving. Interesting as this prospect may be to some, it smacks of an excessively questionable ideology, with limited relevance for public health.

Alternatively, there is no ethical justification in looking for such alternatives. The cell lines continue to exist, they are beneficial, and their origin can be separated from the beneficial effects of vaccines that save millions of lives. The ethical equation pits two sets of values against one another: the value of a small number of destroyed fetuses in the past, the destruction of which was completely beyond our control, against the value of countless lives today that could be saved by the use of a vaccine.

### Vaccines and Saving Lives

Vaccines for varicella, rubella, hepatitis A, rabies and COVID-19 (Johnson & Johnson (J&J)/Janssen) are all made by growing the viruses in fetal cells. All of these, except the COVID-19 vaccine, are made using fibroblast cells. The COVID-19 vaccine (J&J/Janssen) is made using fetal retinal cells. The fetal fibroblast cells used to grow vaccine viruses were first obtained from elective termination of two pregnancies in the early 1960s. These same fetal cells obtained from the early 1960s have continued to grow in the laboratory and are used to make vaccines today. No further sources of fetal cells are needed to make these vaccines. The reasons that fetal cells were originally used included the fact that viruses grow best on human cells. The current fetal cell lines are thousands of generations removed from the original fetal tissue and contain no tissue from the fetus. The RNA vaccines (Pfizer and Moderna) and Novavax’s protein vaccine do not use human cells for production, but they are used to test the effectiveness and safety of the medications. Several vaccines currently available in the United States were developed using animal cell strains, primarily cells from African green monkeys. These include vaccines against Japanese encephalitis, rotavirus, polio and smallpox. Of these, only rotavirus and polio vaccines are routinely given.

Regardless of how a vaccine is obtained, it will still prove extremely effective in saving lives. This does not mean that the ethical issues around abortion should be dismissed, nor that those who are troubled by abortions should cease to advocate for alternatives. But it leaves no room for rejecting the legitimacy of making effective vaccines available as widely as possible. Such vaccines are ethical, even for those who consider their origin in, or association with, abortion to be ‘evil,’ as long as their origin is acknowledged, and there is stringent separation between their origin and contemporary use.

### Moral Justification of COVID Vaccines

The Congregation for the Doctrine of the Faith (2020) has come to the conclusion that use of COVID vaccines is morally licit, if the cooperation in evil (passive material cooperation) is remote and if the vaccines will help contain the spread of a serious pathological agent. According to this argument, all vaccinations can be used in good conscience since their use does not constitute what is described as formal cooperation with the originating abortion. A driving force here is the duty to protect not only one’s own health but also the common good, and in particular, to protect the weakest and most exposed.

Much the same conclusion has been reached by others, albeit employing different terminology. For instance, Robert Orr suggested the following criteria for evaluating moral complicity: timing, proximity, certitude, knowledge and intent (Zimmerman, 2021). Clear separation between the original act and subsequent use of the fetal cell lines once again came to the fore, with emphasis on the beneficial effects of the vaccines, their independence from the abortion, the motive of vaccination being protection of the community and the ultimate goal of saving large numbers of lives.

This last point reveals an inherent moral contradiction for those concerned about abortions, because their vaccine refusal ultimately undermines that for which they are striving – the dignity of human life. How is it possible to contend for fetal life but ignore the far greater number of lives that will be lost by an anti-vaccination stance? The lack of consistency in this position is troubling and ultimately self-defeating (Kleinisman and O’Brien, 2021). While the situation creates a genuine dilemma for some, it is not akin to overlooking what may be considered to be the immorality of abortion. Additionally, given the current danger, being vaccinated against the coronavirus is accurately described as an act that upholds, and is arguably even demanded by, our duty to the common good of society.

In spite of this, some Roman Catholic writers continue to argue that the use of fetal tissue from elective abortions desensitises beneficiaries, scientists and doctors to the original evil act that produced these cells (McKenna, 2018). The aim is to affirm the value of all human life, and yet no attention is paid to the large number of lives lost or severely compromised if efficacious alternative vaccines are unavailable. This looks like an increasingly untenable position that rejects the gratitude to be shown for the many years of work in developing effective vaccines against a host of heart-breaking diseases.

## Freedom of Choice

This represents a move from an area of well recognized ethical debate into relatively opaque territory, dominated by very conservative religious groups for whom vaccine-related concerns emerge out of a plethora of unclear theological trends. While the abortion issue may never be far from the surface, it has become swathed in a network of issues that have come to the fore with the prospect of the introduction of vaccine mandates. Although these objections come from a minority of Christians, the ones espousing them are, at the best vaccine hesitant, if not decidedly anti-vaccine.

While the religious objections proclaimed by conservative Christian groups do not represent the broad sweep of Christian responses, they garner considerable publicity and in doing so have an undue influence on church goers. This phenomenon is most vividly expressed by the views of senior pastors of some large Pentecostal and charismatic churches. An approach commonly encountered is that of opposition to the introduction of vaccine passports, although lying behind this is vaccine hesitancy, even when only subtly expressed. The result is that it is difficult to identify the strands of the argument, since they are hidden beneath a welter of obfuscation.

One frequently hears a pastor state that he refuses to tell anyone whether they should or should not be vaccinated; that is entirely a personal decision. However, the thrust of the message is to throw doubt on vaccines and on those who are advocating for their use. An example is provided by the following statement from one such church: “[The] Church takes no official or ethical stance on the use of vaccines. Neither do we believe that we are in a position to offer medical advice to others. We encourage individuals to make an informed decision based on personal conscience and the counsel of qualified medical professionals” (Mortlock, 2021a). However, the statement goes on to claim that people need to hear all sides of the story and is concerned about the “prevalent, fearful rhetoric from the government and media”. Everyone should, it is stated, have a right to question and challenge what they hear from official sources, and the church strongly objects to any restrictions that would limit in-person church attendance based on vaccination status. The by-line is that everyone has the right to make their own decision in respect of vaccination.

### Lack of Trust in Political and Scientific Experts

However, the tone is set by a medley of complaints about official messages aimed at encouraging vaccination and at the science underlying these messages. A distinct lack of trust in the expertise of scientists, policy makers and politicians comes through, with opposition to government control (Mortlock, 2021b). This is made all the worse because, it is claimed, the government and the media are not letting people know the full truth, with hints that a conspiracy is afoot; for example, there are allegedly many deaths associated with use of the COVID vaccines, and since those opposed to vaccination are being silenced, there is no avenue for open discussion of developments such as these. The main problem it is asserted that is fear mongering, since COVID-related deaths need to be seen in the context of the far greater number of deaths from other causes. Overriding this motley of messages is strident opposition to any restrictions on church attendance and on the increasing restrictions on freedom throughout society (Mortlock, 2021c). This is said to be part of a social engineering project aimed at social control, of which vaccine passports are simply one manifestation. The underlying mantra is that of freedom of choice across society, but with emphasis on freedom to worship.

In reflecting on this stance as an expression of a religious perspective, what emerges is considerable distrust in science, medicine, specialist experts and the elite in general. Even more striking is the lack of specific religious content, with individualism replacing communitarianism, and an emphasis on individual rights rather than the welfare of the weak and susceptible. Running throughout this rhetoric is an inherent distrust of science and scientists, who are portrayed as posing a threat to the moral order.

### Religious Leaders as Part of the Problem

These examples are illustrations of religion and religious leaders as part of the problem, rather than part of the solution (Levin, 2020). This is a tragedy with its

rejection of the centrality of science in understanding and responding to a viral pandemic (Jones, 2020, 2021), and its misleading of the faith community by promulgating misinformation, theological ineptitude and mistrust of all in authority. In this way, it fails to offer hope and direction to its congregants, let alone any sort of pastoral support, and may even be responsible for a worsening health crisis. In an analysis of vaccine hesitancy, Dielschneider (2021) sought to address some of the major objections raised against vaccines from a Christian perspective. For instance, she argued that vaccines, like other medical advances, are a form of divine providence and that they do not defile the body as God’s temple any more than most of the food we eat. For her facts and logic combined with empathy may well constitute the most successful approach to combat vaccine hesitancy. Levin (2020) has commented: “If we allow ourselves to fall victim to charlatanism posing as religious truth, then the outbreak will have been a wasted opportunity for growth, for rethinking why we are here and for reordering our priorities.”

The difficulty here is the call to freedom, that is, freedom from vaccine mandates and to do as one pleases regardless of the consequences for other people. This is the opposite of the notion of the freedom found within Christian thinking, namely, that we are free to serve God and our fellow beings (1 Corinthians 8:9; 9:12-23; 10:23-33). Christian freedom lies in being able to assess the risks of COVID, the effectiveness of vaccines, the frequency of hospitalizations and deaths with and without vaccination, and the ethical arguments justifying the use of the available vaccines (Piper, 2021). It is in taking account of all this evidence that Christians can live as free people in Christ, doing what they know is best for them and others, regardless of what any authorities or conspiracy theorists may state.

## Vaccine mandates

The possibility that vaccine mandates will be required for entry into a range of venues or workplaces has been vigorously opposed by a range of Christian church leaders in a number of countries. It is this opposition that has elicited a great deal of public attention, to the exclusion of far more moderate responses from other churches. It is fitting, therefore, to look initially at these more extreme stances.

In an open letter to the UK prime minister, three sets of reasons were outlined (Philip et al., 2021). First, these leaders argue that proof of immunity makes no sense in terms of protecting others. Second, vaccine passports would constitute an unethical form of coercion and violation of the principle of informed consent. Third, they risk creating a two-tier society, described by them as ‘medical apartheid’ in which an underclass of people will be excluded from important areas of public life. The writers envisage this scheme having the potential to bring about the end of liberal democracy and the creation of a surveillance state. Their opposition to any form of vaccine passport has particular relevance to church attendance. They write: “to shut out those deemed by the state to be social undesirables would be anathema to us and a denial of the truth of the Gospel.” (Philip et al., 2021). For them, this is an illiberal and dangerous plan.

Similar opposition to vaccine passports is present in Australia with open letters to the prime minister under the banner of the Ezekiel Declaration (2021) and the Moses Statement (2021). The Ezekiel Declaration, written by three Baptist pastors, regards a vaccine passport as a form of ‘therapeutic totalitarianism’, with what the writers see as its potential to dehumanise and control citizens on the grounds of personal health and safety. An underlying concern appears to be that the government is coercing people’s conscience, especially as it is claimed some may have valid reasons for being hesitant about vaccination. While these concerns are all of a general nature, they also find it untenable that people would be refused entry into churches on the basis of their medical choice, and hence unable to hear the message of the Christian gospel. This latter point is taken up far more specifically by the writers (four Presbyterian and one Baptist ministers) of the Moses Statement, who address the prime minister and state premiers in far more direct biblical terms, claiming that Christians are to meet together physically for worship and that “no government on earth should tell the church it cannot gather to worship” (Moses Statement, 2021).

Taken together these two documents have been signed by thousands of religious leaders from a wide range of churches in Australia. Both appear on the same web site, Caldron Pool (an Australian conservative news website named after a lake in CS Lewis’s Narnia chronicles).

### Divergence of Viewpoints

It is interesting that these statements, despite their Old Testament allusions in their names, do not represent the views of all churches or Christian groups in Australia. For instance, the Ezekiel Declaration is critiqued by one group of Presbyterians for its misleading claims about vaccines (Gospel, Society & Culture, 2021). While sharing some of the social concerns about vaccine passports, they do not think that most objections to COVID passports are a matter of conscience nor that a passport scheme need be coercive. Even more, these writers criticize what they take to be the highly charged language of the document, with its confusion of lockdowns and passports and its general tenor of suspicion of the government. In a response to criticisms of this nature, the writers of the Ezekiel Declaration make clear that they see their opposition to vaccine mandates as an outworking of their calling to be faithful followers of Jesus and as good citizens (Staff Writer, 2021). Hence, although the content of the Declaration is far from explicit (unlike the Moses Statement), the opposition to vaccine mandates has a religious basis.

Other religious commentators have interpreted the statements emanating from the Caldron Pool as promulgating the view that Christianity is the natural bedrock of a nation. In this context, it is easy to see that those restrictions will be interpreted as an encroachment by the state on the authority of the church (Sheperd, 2021). This stance is made possible by a libertarian and individualist streak, generally not evident in mainstream Christian thinking. It also throws doubt on lockdowns and the central significance of vaccination as crucial to public health measures. The absence of any emphasis on doing what is best for the community is strange coming from those advocating for a Christian response grounded as that usually is with love for one’s neighbour and protection of the vulnerable and the dispossessed.

### Conscience and Coercion

In further missives from the Caldron Pool, stress is laid on conscience and coercion. Chan writes: “Refusal to have a vaccine may be motivated by a principled decision to resist therapeutic coercion and compulsion, the segregation of society based on vaccine status, and the dangerous growth of State and employer power. It may be motivated by a desire to stand against a consequentialist ‘ends justifies the means’ ethic which permits the evil of coercion to achieve the good of reducing disease burden. These are all moral matters, and therefore matters of conscience” (Chan, 2021). In Christian terms, “widespread coercion of vaccination [is] an assault on the Kingship and Lordship of Jesus Christ.”

Underlying these concerns is vaccine hesitancy, leading to the view that vaccination is a therapeutic invasion of one’s body using a risky substance, allied with rejection of any form of coercion even for good ends (the saving of life). His conclusion is that these are matters of conscience and the right to decline vaccination without suffering penalties should be fiercely defended by Christians (Chan, 2021). These are, in his estimation, significant human rights violations, and yet no serious attention is paid to the negative health consequences for the community, including the vulnerable and disadvantaged. Neither is there any recognition that, even without any form of coercion, society will end up being segregated on health grounds, between those in a position to protect themselves from a virulent pandemic, and those lacking the opportunity to do so. Chan’s position demonstrates a concerning lack of love for those unable to protect their own interests.

### Christian Values

A far more nuanced approach to vaccine passports has been provided by the Church of England, based in part on documents provided by the Royal Society and the Ada Lovelace Institute (Church of England, 2021). Ethical issues considered foundational in approaching vaccine passports touch on public health considerations, personal liberty, economic benefits and future risks. Under these headings, arguments for and against passports are outlined, with further thought being given to those unable to be vaccinated on physical or mental health grounds and who might consequently be disadvantaged, and threats to the notion of fully informed consent. Additionally, threats to the freedom of religious belief were noted if objections based on the use of aborted fetal cells or testing on animals were ignored.

In applying Christian values to this debate, the following were highlighted: identification with the poor and marginalized, respect for individuals’ dignity, belief in grace and mercy rather than judgement, and commitment to equality. In light of principles such as these, the Church of England encourages individuals to be vaccinated, both for their own sake and for the sake of others, but considers that vaccine passports should not receive the same high-level endorsement. It concludes: “While the Church is, in principle, opposed to making use of ‘vaccine passports’, it should adopt a flexible approach to their *limited* wider use with the important caveats that such use ought to be demonstrably beneficial to society as a whole, protective of the vulnerable in particular, non-discriminatory in nature and proportionate in use” (Church of England, 2021).

This attempt to balance encouragement to be vaccinated against limiting the use of vaccine passports to situations where it can be justified on the basis of clear ethical principles requires discernment on the part of both government and individual businesses. The Church’s position is determined by the foundational principle that the Church is a home and refuge for all, meaning that the Church itself would only wish to utilize vaccine passports in exceptional circumstances. To what degree these delicate balancing acts can be operationalized in society will depend on extant government regulations in different locations and countries.

Acceptance of the principle of COVID vaccination is common among most mainstream Christian churches and groups on the ground that this is a means of disease prevention. The benefits of prevention extend beyond oneself and help protect the local and global community. Interestingly, this position is very clearly expressed by the Seventh Day Adventist Church, with its emphasis on holistic health measures (General Conference of Seventh-day Adventists, 2021). They are not opposed to public safety and government health mandates, although they stress that they accept that members on occasion will have personal concerns and conscientious convictions that go beyond the teachings of the Church. But where there are disagreements, the Church stresses the need to relate to each other with respect, love and compassion. Love for neighbour shines through, both in preventing the spread of deadly disease and in the manner in which individuals relate to each other.

### Protection of the Community

The major thrust of mainline Christian responses is that vaccination protects not only the individual, but the individual’s community. This immediately redirects the spotlight from the individual onto the community (Best, 2021). The core position is, therefore, in favour of vaccination rather than against it. And so, when vaccine mandates are under consideration, and freedom to attend church is being debated, the importance of vaccination is not downplayed. This means that care is required in working out how best to apply biblical passages that refer to the early church and determine their relevance for the contemporary Christian world, especially that of very large churches.

The significance of respecting the consciences of others, leads to exercising restraint in inflicting vaccination on them. And yet, as Best writes: “conscientious objection usually comes at a cost of some sort and the safety of the most vulnerable members of the church and the wider community should not be held hostage to the desire of others to do as they please, irrespective of government health orders or the risk to others” (Best, 2021). Alongside this principle, goes that of prioritizing the weakest and most vulnerable, especially those with poor health and lack of a public voice, as much as those strongly opposed to vaccination. Best concludes: “With care, creativity and a willingness to pursue the good of others ahead of our own convenience and advantage, it should be entirely possible for us to practise *both* our call to minister the gospel to all people *and* our responsibility to love our neighbours and care for the vulnerable, without requiring one of these commitments to trump the other” (Best, 2021).

The Vatican, having determined that the use of anti-COVID vaccines is morally acceptable (Congregation for the Doctrine of the Faith, 2020), has come out in favour of vaccine mandates. All Vatican employees have to show proof of vaccination, a negative test, or have recently recovered from COVID. The Pope has repeatedly encouraged vaccination and refers to vaccines as “an act of love.” For the Pope, “Vaccination is a simple but profound way of promoting the common good and caring for each other, especially the most vulnerable” (Jenkins, 2021; Watkins, 2021).

### Public Worship

This still leaves the crucial question how churches are to organize their public worship during a pandemic. What is the nature of the decision-making and on what values is it to be based? The specifics will undoubtedly vary between places, depending upon the regulations imposed by government authorities. In the earlier stages of the pandemic, during lockdowns, major concerns were with the manner in which worship practices were being affected, community transmission, social distancing and the welfare and pastoral concerns of church congregations (Oxholm et al., 2021).

The major concern of churches is that no minority within them will be excluded let alone vilified. The group in question is usually the non-vaccinated, since they are the ones who have raised objections to mandates and to the restrictions being imposed upon their freedom to worship. Others will be concerned about those outside the churches who may be kept from hearing the claims of the gospel of Jesus Christ. The major thrust generally though is protection of the vaccinated and especially those who for health reasons are in a vulnerable place. This creates division, but there is no escape from division, for as long as there are two populations, namely the vaccinated and the unvaccinated. The challenge for churches is to decide how best to approach the two groups in a manner that will aim to understand the other perspective, based on grace and love to all.

Depending on the regulations and the size of the church, churches may have a single service open to all, a single service limited to the vaccinated, or two services - one open to all and one limited to the vaccinated.

## Dissecting Mandates

Mandates can only be justified if there are strong grounds for justifying vaccination itself. If this is not the case, the ethical basis for vaccine mandates disappears. Hence, the fundamental premise is that vaccines are safe and effective (see Safety and efficacy of the COVID vaccines).

The fundamental equation involves weighing up the benefits and harms of both vaccination and vaccine mandates. For those in favour of vaccinations, the benefits to the community substantially outweigh any harms to the individual; the vaccine hesitant consider that potential harm to the individual overrides any prospective benefits to the community. The issue revolves around the scientific evidence on the respective benefits and harms in both cases. Since the conventional scientific position is that the benefits of vaccines are considerable, the only way of rejecting this position is to reject conventional scientific explanations.

Vaccination is by far the most effective way of combatting the worst excesses of the COVID pandemic. However, since some are reluctant to accept this course of action, a way has to be found of increasing the rates of vaccination in the population. This is where mandates appear as a last resort. Education is the preferred pathway, but when education is rejected or ignored and voluntary vaccination fails, some form of coercion is resorted to.

Some argue that this is unethical, since it is not based on informed consent, and involves introducing a foreign substance into the individual’s body against their wishes. However, this fails to take account of the balance between harms and benefits. Since vaccination and mandates aim to protect the health of individuals and of the community, and for which there is overwhelming evidence of their success, there is justification for restricting the actions of the vaccine hesitant. This is because they have been given ample opportunity to be vaccinated, have been informed of the justification for vaccination, and the harm caused by the lack of vaccination for the community. Under these circumstances, coercion can be justified as long as it is proportionate and necessary to maintain public health. In principle, the benefits outweigh the harms to the community. Nevertheless, mandates have to be imposed with care, since they restrict the freedom of individuals. Therefore, they can be justified only when they are the least invasive, least restrictive alternative reasonably available, and likely to be effective to achieve the goal of minimizing the spread of COVID-19.

### Policy Options

Any restriction on people’s movements has ethical repercussions (Hall & Studdart, 2021). The Association of Bioethics Program Directors has no quibbles on the matter (Association of Program Directors, 2021; Caplan, 2021). For them, compulsion is necessary since this is a very serious public health emergency. Not only this, the Association argues that it is not really compulsion since people are still free to make choices, although they do not spell out what these choices are. They reject the notion that a mandate violates rights to individual liberty and autonomy, since they prevent risk to others, including disabling complications, psychosocial havoc and burdens on health systems.

Others are not as definitive as this, pointing to the low rates of vaccination among racial minorities and low-income populations, and any privileging of the vaccinated will penalize people with religious and philosophical objections to vaccination (Hall & Studdart, 2021). The phenomenon of low vaccination rates among indigenous groups is very common, due to long-standing negative experiences of the dominant health sector and aggravated by misinformation and disinformation within their communities. Concerted efforts are required to address this situation by prioritizing vaccination for these groups, utilizing the health and cultural expertise of members of their own communities.

These concerns are salutary, but they fit within a range of policy options, the extremes of which are a broad mandatory public scheme at one end, and prohibition of all private uses of certification at the other. Distinctions have to be made between their use in different contexts, from international travel, to teaching in educational settings, and the health system; and from attendance at sports events, concerts, clubs, restaurants and bars (Hall & Studdart, 2021). Some of these are public events, under the aegis of government bodies and with consequences for the health and wellbeing of the general public. Others are private functions, which people are free to attend for their own benefit or amusement. The distinction here is between a regulatory environment where evidence of vaccination could/should be mandated, and the non-vaccinated are not allowed access, and a more flexible regime where there is some latitude to set rules by those in charge of the venues. However, in this latter case, care will be required to ensure there is not inequity and that evidence is presented of being COVID-free.

### Mandate Exemptions

In all cases, exemptions will have to be made for those unable to be vaccinated on health grounds, or other as yet undetermined reasons. Highly contentious as these grounds will prove, and unfortunately open to abuse, it will be important to build flexibility into whatever systems are put in place. Only in this way will the complexity of COVID-19 be managed, as well as its rapidly changing dimensions. It is notable that the WHO has advised against the implementation of immunity certification at present on the grounds that there is uncertainty that long-term immunity exists and concerns over reliability of serological test methods for determining immunity (Voo, 2021). There is also concern over restrictions imposing disproportionate burdens on non-certified individuals and violation of individual liberties and rights. However, the major consideration is that immunity certification should not be used as the main strategy for reducing the effects of the pandemic, but should be imposed as a component of a plan that decreases the number of people subject to very restrictive measures and increases those able to take on higher-risk activities like caring for others and providing services for others (Harper, 2021).

Mandates aim to benefit the health system. This is the benefit that is normally highlighted, namely, protection of the integrity of the health system. The goal here is to protect the functioning of hospitals and the interests of their staff. If the latter are laid off or die this will have widespread detrimental effects on many others in the community, who suffer from COVID, as well as those who will miss out on routine treatment for non-COVID-related conditions.

Less attention is generally paid to any social consequences for the unvaccinated, such as not being allowed to take part in a range of regular social activities, leading possibly to regarding themselves as social outcasts. This can be regarded as a harm, even though the unvaccinated are in this position as a consequence of their own choice. While they have not chosen to be ostracized, they have acted in a way that they knew would lead to some degree of exclusion from regular social activities.

### Churches and Vaccine Mandates

In view of these considerations what role do vaccine mandates have to play in church circles? The onus is on protection of the vulnerable, including unvaccinated children and the immunocompromised. All the evidence discussed above points towards churches having vaccine mandates as a means of protecting the community as a whole. However, the arguments for allowing everyone to attend church services and receive the religious sustenance they have to offer are compelling. In order to meld these two directives, there appears to be an onus on those who have refused vaccination to have to think through the consequences of their decision on other members of the community. If they are allowed to attend large gatherings regardless of vaccination status, it is incumbent upon them to provide evidence of negative COVID tests. Regardless of their reasons for rejecting vaccination, this is a reasonable stricture to place upon them to protect their fellow attendees. It also reminds them that a dominant value for most religious groups is the good of their fellow human beings; in religious language love for one’s neighbour as a determinative driver of ethical behaviour (Mark 12: 30–31).

Regardless of what decisions have been taken, it is impossible in a pandemic for people to be treated in a uniform manner; if all services are open to all, some vaccinated people will not attend out of fear of being infected; if there are ‘closed’ and ‘open’ services (regardless of their designation), for the vaccinated and for both vaccinated and unvaccinated, respectively, all will not be able to meet together. A decision has to be taken. If church leaders remain on the fence, they are relinquishing their authority and are exposing church adherents to unnecessary risks. The church leadership should play a role in stressing that, even in the middle of a pandemic, the one and only focal point is the Lordship of Christ and not a vaccine or any other subsidiary emphasis.

## Back to Science and Faith

A major difficulty in the COVID vaccination debate is the apparent inability of some of those coming from a religious background to distinguish scientific evidence from theological and cultural perspectives. While it is generally unhelpful to regard these two realms as separate watertight compartments, the COVID situation has introduced strange anomalies. Many Christians objecting to COVID vaccines do so on scientific grounds, such as the experimental nature of the vaccines, and their alleged deleterious side-effects, or on cultural grounds such as the increasing power of governments or the duplicity of government officials and scientists. The theological grounds tend to be far less prominent, except for their postulated link to past abortions. Other issues can only be described as ephemeral, such as the claim that God will protect us from viruses, and that our bodies should not be modified by vaccines.

The lesson to emerge from this is that far more attention needs to be paid to the scientific realm, with far greater stress being placed on the wellbeing of the vaccinated who are the ones responding to the goodness of God displayed in the creativity and ingenuity that has led to the production of remarkably effective vaccines. Thankfulness and gratitude to God by the people of God would be a far more fitting response to vaccines and vaccine mandates, together with an urgent drive to make vaccines as available as possible worldwide. The lack of even the merest hint of vaccine equity across the world is a blot on the moral landscape, and while this is beyond the ability of individuals or even individual church bodies to rectify, it is imperative that churches in the West make their voices heard in advocating vigorously for those less favoured than themselves. This should be the heart and impetus of any religious response to the current pandemic.

## Limitations of the Study

Emphasis has been placed on the more conservative elements within Protestant churches, with less attention on other traditions. There has also been a concentration on responses by those churches within Australia and New Zealand influenced by American theological trends. Inevitably, the study reflects discussions and deliberations occurring in a limited time span, 2020-early 2022.
